# Frequent Canned Food Use is Positively Associated with Nutrient-Dense Food Group Consumption and Higher Nutrient Intakes in US Children and Adults

**DOI:** 10.3390/nu7075240

**Published:** 2015-07-09

**Authors:** Kevin B. Comerford

**Affiliations:** 1 Department of Nutrition, University of California at Davis, Davis, CA 95616, USA; E-Mail: kbcomerford@ucdavis.edu; Tel.: +1-707-799-0699; 2 OMNI Nutrition Science, Sacramento, CA 95819, USA

**Keywords:** canned food, food groups, nutrient-dense, nutrient intake, diet quality

## Abstract

In addition to fresh foods, many canned foods also provide nutrient-dense dietary options, often at a lower price, with longer storage potential. The aim of this study was to compare nutrient-dense food group intake and nutrient intake between different levels of canned food consumption in the US. Consumption data were collected for this cross-sectional study from 9761 American canned food consumers (aged two years and older) from The NPD Group’s National Eating Trends^®^ (NET^®^) database during 2011–2013; and the data were assessed using The NPD Group’s Nutrient Intake Database. Canned food consumers were placed into three groups: Frequent Can Users (≥6 canned items/week); *n* = 2584, Average Can Users (3–5 canned items/week); *n* = 4445, and Infrequent Can Users (≤2 canned items/week); *n* = 2732. The results provide evidence that Frequent Can Users consume more nutrient-dense food groups such as fruits, vegetables, dairy products, and protein-rich foods, and also have higher intakes of 17 essential nutrients including the shortfall nutrients—potassium, calcium and fiber—when compared to Infrequent Can Users. Therefore, in addition to fresh foods, diets higher in nutrient-dense canned food consumption can also offer dietary options which improve nutrient intakes and the overall diet quality of Americans.

## 1. Introduction

Proper nutrition is one of the most effective and least expensive ways to prevent and treat several chronic- and deficiency-related diseases [1]. A key component to proper nutrition entails finding and maintaining a healthy balance between calorie and nutrient intake. However, the ability to achieve a balance between calories and nutrients differs for individuals and populations across the globe, depending on a multiplicity of genetic, lifestyle and socioeconomic factors. The federal dietary guidance for industrially developed and westernized populations such as the general population in the US includes recommendations to reduce overall calorie intake (especially from added sugars and solid fats) and to increase intake of nutrient-dense foods such as vegetables, fruits, unsalted nuts and seeds, beans and peas, low-fat dairy, lean meats, and seafood [2]. All of these recommended foods, with the exception of dairy products, nuts, and seeds are commonly consumed in both fresh and canned varieties.

Canned foods are a core component of the diets of many Americans. A 2013 survey of more than 1000 Americans showed that greater than 60% of the respondents reported using canned foods at least once or twice each week [3]. Still, many Americans use these products for their cost and/or convenience; and not for their nutritional properties [3]. In general, fresh foods are recommended as the primary nutrient-dense dietary options, but fresh foods are not always available to all Americans due to seasonal, economic and geographic factors. Recent research has shown that canned options contain comparable nutrient profiles to fresh foods [4,5,6,7], therefore nutrient-dense canned foods should be considered as healthy options alongside fresh foods, or as nutritious alternatives to fresh foods. Additionally, a study by Kapica *et al.*, provided evidence that canned foods are some of the most cost-effective and accessible nutrient options available to Americans [5]. Furthermore, the 2010 Dietary Guidelines for Americans (DGA) promotes nutrient-dense canned foods such as vegetables, fruits, legumes and seafood (especially those which are low in salt and sugar) along with fresh and frozen options to meet the recommended dietary intakes for food groups and nutrient levels [2].

Although canned foods are featured in grocery stores, and used in restaurant meals and government assistance programs, the frequency of canned food consumption in the US has been on a steady decline over the last 10 years with Annual Eatings per Capita (AEPC) rates decreasing by 7.5% from 2003 to 2013. The “eat rate” (*i.e.*, the average number of times a using individual consumes a product during a two-week period) for canned foods has also declined by 4% over the same time course [8]. The misconceptions about the nutritional impact and healthfulness of canned foods, and their decreasing usage rate may both be contributing factors to the nutritional inadequacy of the American diet. The objective of this study was to compare and contrast the dietary intake and nutritional profiles of canned food consumers based on their frequency of canned food use to better understand the role that canned foods play in the American diet. This is the first study of its kind to compare food group intake and nutrient intake in US children and adults based on the frequency of canned food consumption.

## 2. Experimental Section

### 2.1. Sampling and Data Collection

The National Eating Trends (NET^®^) database has continuously tracked representative sample of adults’ and children’s total diet consumption for 30 years and is conducted year round to be able to account for changes in weekly and seasonal eating behaviors. For this study, data were collected annually from 2500 US free-living households in NPD NET^®^ database and NPD’s Nutrient Intake Database over the course of two years (February 2011 to February 2013). NPD received ethical approval for this study from the Council of American Survey Research Organizations (CASRO) and adhered to the mandated CASRO Code of Standards and Ethics for Survey Research. The NET^®^ database is a nationally representative sample using a stratified quota sample of present demographic composition to meet U.S. Census Bureau demographic targets. Its main purpose is to capture all foods and beverages consumed by each family member in a household, both in home and away from home, during 14 consecutive days each year. NPD’s Nutrient Intake Database used in this study provides estimates of daily intake at the individual level for calories, macronutrients and micronutrients. The database calculates nutrient intake data by integrating the eating frequency from the NET^®^ database with average serving sizes from the What We Eat in America (WWEIA) dietary intake interview component of the National Health and Nutrition Examination Survey (NHANES) and nutrient values from the United States Department of Agriculture’s (USDA) National Nutrient Database for Standard Reference.

Panelists were recruited from a national mail panel to participate in NPD’s NET^®^ survey. Candidate panelists received a sample diary, instructions, and one actual daily diary to complete and return. Only panelists returning acceptable diaries were asked to participate in the 14-day study. Each panelist was responsible for the food records for their entire household. The individual sample was therefore comprised of all household members from participating households. All households had to pass quality control checks on the completeness of their food diary reporting. Households had to return at least 10 of 14 diaries to be considered eligible for the quarterly sample. Each diary captured all foods and beverages consumed in-home, carried-from-home, and away-from-home, in separate sections. Meal occasions were identified as main meals or snacks. Information collected included detailed food descriptions, including brand names, preparation methods, and appliances used.

### 2.2. Analysis

Food diary data were initially analyzed from 2000 households per year (*n* = 4000 households total), resulting in food diaries from approximately 5000 individuals per year (*n* = 10,000 total), and over 200,000 eating occasions annually (*n* > 400,000 eating occasions total). The final analysis focused on individuals two years and older (*n* = 9761). Depending on their consumption habits, canned food consumers were placed into three groups: Frequent Can Users (FCU); *n* = 2584, Average Can Users (ACU); *n* = 4445, and Infrequent Can Users (ICU); *n* = 2732. FCU were defined as those individuals who consumed canned foods six or more times in the two weeks study period, while ICU consumed canned foods 1–2 times over the two-week study period. ACU consumed canned goods between three to five times during the two-week study period, and were not included in the final analysis. Two groups were singled out and dichotomized for further investigation and comparison of canned food consumption—infrequent users relative to frequent users. Additional analysis was performed to determine the overall and subcomponent diet quality on selected days. Specifically, to compare essential nutrient intake on days when canned foods were eaten *versus* days when canned foods were not eaten.

Data extraction and analysis of food and nutrient intake was performed on the NPD’s NET^®^ intake diary panel and Nutrient Intake databases. Foods were placed into categories such as “Canned,” “Ready-to-Eat (RTE),” “Homemade,” “Fresh,” “Frozen,” and “Refrigerated.” Canned foods included base dish/additive canned foods (*i.e.*, processed fruit, processed vegetables/legumes, finfish, soup, processed meat and combination dishes that are canned or in aluminum/metal packaging). Consumption data were used to estimate canned food eating occasions over the two-week period.

For each variable, estimates of the mean were given, as were standard deviations where appropriate. A 95% confidence interval (95% CI) was used for the mean. Two-tailed *t*-tests were used and the significance was set at CI > 97.5%, (or *p* ≤ 0.025). Tests of significance were completed for participants who were frequent can users and for participants who were infrequent can users. NPD’s Nutrient Intake data was processed in SAS software version 9.3 (2011, SAS Institute, Cary, NC, USA).

## *3.* Results

### 3.1. Study Demographics and Sample Sizes

The final sample consisted of 5316 survey respondents—1165 children and adolescents between the ages of 2–17 years and 4151 adults (64% female, 36% male) over the age of 18 years. The ACU group (*i.e.*, those who consumed 3–5 cans in the two-week study period) and children under two years of age were not included in the final analysis. The data from children over the age of two years, adolescents and adults were merged and analyzed together in order to focus on the dietary and nutritional differences between frequent and infrequent canned food users in the general American population. The FCU group (*n* = 2584) consumed canned food six or more times in the two-week study period while the ICU group (*n* = 2732) consumed canned food two or less times in the two-week study period. There were no significant differences in age, gender or body mass index (BMI) between the FCU and ICU groups (Table 1). The percentage of frequent and infrequent canned food users was similar in households earning between $10,000 and $70,000 per year, while, households with earnings over $70,000 per year was comprised of a higher percentage of infrequent can users (31.2%) than frequent users (23.2%) (Table 1). The FCU group was significantly more likely to participate in the Supplemental Nutrition Assistance Program (SNAP) government assistance program than the ICU group; and almost twice as likely to participate in the Women, Infants and Children (WIC) program, although this difference did not reach statistical significance (Table 1). When combined, there were approximately 40,000 instances of canned food consumption during the two-week period between the two groups, with the FCU group accounting for approximately 2/3rds of the total consumption of canned foods.

**Table 1:** Demographics of frequent and infrequent canned food users.

(ICU, *n* = 2732; FCU, *n* = 2584)			
Demographics	% Infrequent Can Users (ICU)	% Frequent Can Users (FCU)	*p*-Value
Age (years)			
2–17	20.5	23.4	NS
18–65	65.6	59.6	NS
65+	13.9	17.0	NS
BMI			
Underweight	4.2	3.8	NS
Optimal	38.2	36.6	NS
Overweight	28.7	32.1	NS
Obese	28.8	27.4	NS
Household Income			
Under $10,000	6.1	10.0	NS
$10,000–$19,999	7.8	11.8	NS
$20,000–$29,999	11.8	17.3	NS
$30,000–$39,999	13.9	14.3	NS
$40,000–$49,999	10.5	12.2	NS
$50,000–$59,999	9.0	5.7	NS
$60,000–$69,999	9.6	5.5	NS
$70,000 and Over	31.2	23.2	<0.01
Assistance Programs			
SNAP participant	11.9	23.5	<0.01
WIC participant	6.3	11.8	NS

Abbreviations: Body Mass Index (BMI), Supplemental Nutrition Assistance Program (SNAP), Woman, Infants and Children (WIC); Percentage of infrequent (*n* = 2732) and frequent (*n* = 2584) canned food users socio-demographic and anthropometric information. Data from the NPD Group’s National Eating Trends intake diary panel 2011–2013 and Nutrient Intake databases. Statistical confidence level set at 95% for two-tailed *t* test. *p*-Value < 0.025% considered significant.

### 3.2. Food Group Intake and Eat Rate

**Figure 1:**
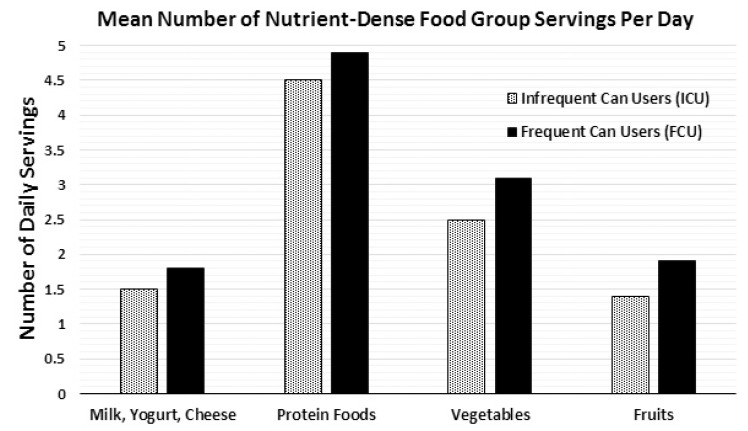
Mean number of nutrient-dense food group servings per day among frequent and infrequent canned food users (FCU and ICU). Proportion of infrequent (*n* = 2732) and frequent (*n* = 2584) canned food users average daily intake of nutrient-dense food groups. Data from The NPD Group’s National Eating Trends^®^ intake diary panel 2011–2013 and Nutrient Intake databases. Statistical confidence level set at 95% for two-tailed *t* test. *p*-Value < 0.025% considered significant. The FCU group had significantly higher values for all food groups listed (*p* < 0.01).

**Figure 2:**
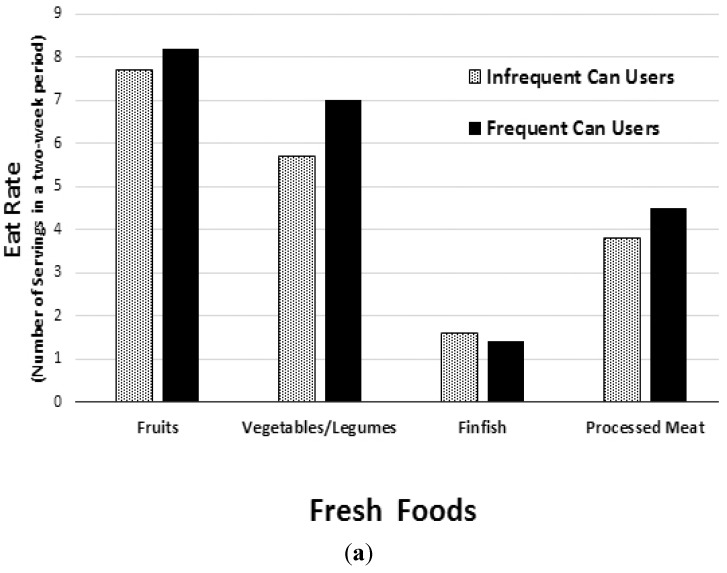

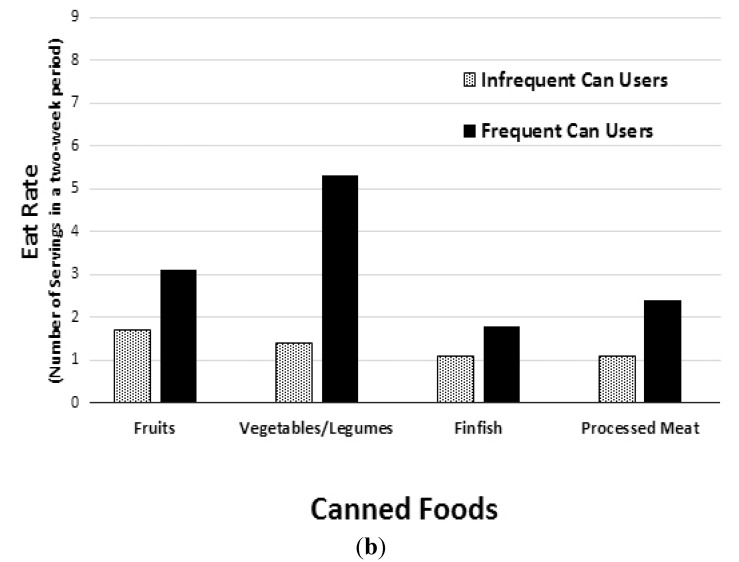
Fresh (**a**) and canned food (**b**) Eat Rate (*i.e.*, average number of food category eating occasions over a two-week period) among Frequent and Infrequent Canned Food Users (FCU and ICU). Proportion of infrequent (*n* = 2732) and frequent (*n* = 2584) canned food users Eat Rate. Data from the NPD Group’s National Eating Trends^®^ intake diary panel 2011–2013 and Nutrient Intake databases. Statistical confidence level set at 95% for two-tailed *t* test. *p*-Value < 0.025% considered significant. The FCU group had a significantly higher Eat Rate for all food groups listed (*p* < 0.01), except for fresh finfish, which was significantly higher in the ICU group (*p* < 0.01).

A comparison of food group intake between infrequent and frequent canned food consumers showed that the FCU group had significantly higher average daily intake of the four primary nutrient-dense food groups; fruits, vegetables, dairy and protein foods (*p* < 0.01) (Figure 1). Compared to the ICU group, the FCU consumed 30.3% more servings of fruit, 21.4% more servings of vegetables, 18.2% more servings of milk, yogurt and cheese, and 8.5% more servings of protein-rich foods such as meat, fish, poultry, beans, eggs, and nuts (Figure 1). Over the course of two week study period, the average Eat Rate was also recorded for fruits, vegetables/legumes, finfish, processed meat and combination dishes (Figure 2). Eat Rate is defined as the average number of times a using individual consumes a product during a two-week period. When compared to the ICU group, the FCU group had a 116% higher Eat Rate of canned vegetables/legumes, and a 58% higher Eat Rate of canned fruits (*p* < 0.01). Interestingly, when compared to the ICU group, the FCU group also had a 20% higher Eat Rate of fresh vegetables/legumes, and 6% higher Eat Rate of fresh fruits (*p* < 0.01). The only category of food in which the ICU group had a significantly higher Eat Rate compared to the FCU group was fresh finfish (13% higher for the ICU group), while the ICU group had a 48% lower Eat Rate of canned finfish (*p* < 0.01).

### 3.3. Nutrient Intake

**Figure 3:**
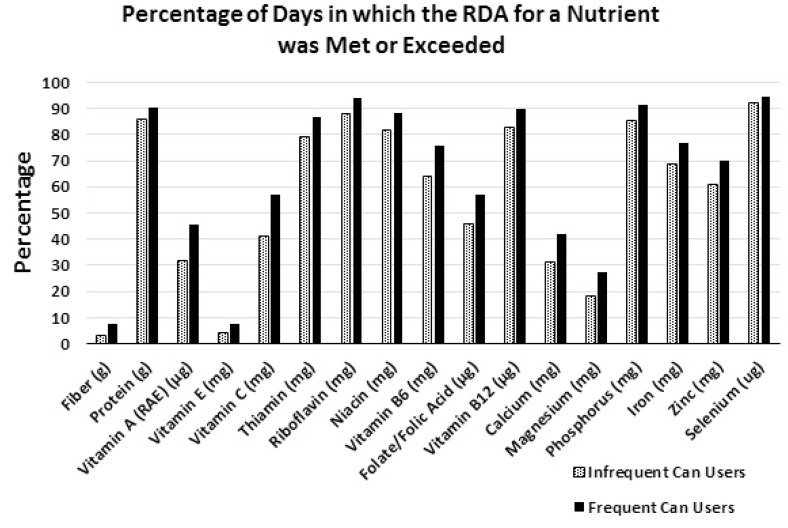
Percentage of days in which participants met or exceeded the Recommended Dietary Allowance (RDA) for different nutrients among Frequent and Infrequent Canned Food Users (FCU and ICU). Percentage of infrequent (*n* = 2732) and frequent (*n* = 2584) canned food users survey days in which the RDA was met or exceeded for a nutrient. Data from the NPD Group’s National Eating Trends^®^ intake diary panel 2011–2013 and Nutrient Intake databases. Statistical confidence level set at 95% for two-tailed *t* test. *p*-Value < 0.025% considered significant. The FCU group had significantly higher values for all nutrients listed (*p* ≤ 0.02).

The FCU group took in significantly higher amounts of 18 essential nutrients and fibers as well, when compared to the ICU group (Table 2). On average, the FCU group took in approximately 3 g more fiber (~20% more), 154 mg more calcium (~16% more), 38 mg more magnesium (~16% more), 24 mg more vitamin C (~31% more), 147 µg more vitamin A (~22% more), and 49 µg more folate (~12% more) each day (Table 2). The FCU group also took in an average of 454 mg more potassium (~19% more), and 420 mg more sodium (~12% more) per day than the ICU group (Table 2). As shown in Figure 3, the FCU group met or exceeded the Recommended Dietary Allowance (RDA) for 16 different nutrients and fibers on significantly more days during the two-week study than the ICU group. Compared with the ICU group, the FCU group had a significantly higher percentage of days meeting or exceeding the RDA for protein (85.8% *vs.* 90.5%, respectively), and fiber (3.1% *vs.* 7.7%, respectively). Compared to the ICU group, the FCU group also had a higher percentage of days meeting or exceeding the RDA for the fat-soluble vitamins, vitamin A (31.8% *vs.* 45.7%, respectively), and vitamin E (4.1% *vs.* 7.6%, respectively). The FCU group met or exceeded the RDA for seven water-soluble vitamins (vitamin C, thiamin, riboflavin, niacin, vitamin B6, folate, and vitamin B12). The RDA for three of the water-soluble vitamins (vitamin C, vitamin B6, and folate) was met or exceeded by an average of over 10% more often during the two-week study period by the FCU group, when compared to the ICU group. Furthermore, the FCU group met or exceeded the RDA for six minerals (calcium, magnesium, phosphorus, iron, zinc, and selenium), with the RDA for calcium, magnesium and zinc being met or exceeded by an average of over 9% more often during the two-week study period, compared to the ICU group.

**Table 2:** Average daily nutrient intake between frequent and infrequent can users.

Frequent Can Users: (ICU, *n* = 2732; FCU, *n* = 2584)			
Nutrient	ICU	FCU	*p*-Value
Fiber (g)	13.1 ± 6.2	16.0 ± 7.4	<0.01
Protein (g)	69.7 ± 26.7	76.3 ± 29.9	<0.01
Vitamin A (RAE) (µg)	585 ± 417	732 ± 417	<0.01
Vitamin E (mg)	6.1 ± 3.5	7.1 ± 4.0	<0.01
Vitamin C (mg)	65.9 ± 64.6	89.7 ± 68.9	<0.01
Thiamin (mg)	1.6 ± 0.7	1.8 ± 0.8	<0.01
Riboflavin (mg)	1.9 ± 0.8	2.2 ± 0.9	<0.01
Niacin (mg)	20.9 ± 8.9	23.2 ± 9.7	<0.01
Vitamin B6 (mg)	1.6 ± 0.8	1.9 ± 0.9	<0.01
Folate/Folic Acid (µg)	370 ± 188	419 ± 200	<0.01
Vitamin B12 (µg)	4.8 ± 4.2	5.7 ± 5.2	<0.01
Calcium (mg)	873 ± 417	1027 ± 493	<0.01
Magnesium (mg)	219 ± 87	257 ± 101	<0.01
Phosphorus (mg)	1178 ± 437	1331 ± 522	<0.01
Iron (mg)	14.3 ± 7.1	16.4 ± 7.8	<0.01
Zinc (mg)	10.4 ± 5.4	11.6 ± 5.8	<0.01
Selenium (µg)	101 ± 44	108 ± 47	<0.01
Sodium (mg)	3168 ± 1283	3588 ± 1482	<0.01
Potassium (mg)	2132 ± 827	2586 ± 988	<0.01

Average nutrient intake (mean and standard deviation) of infrequent (*n* = 2732) and frequent (*n* = 2584) canned food users. Data from the NPD Group’s National Eating Trends^®^ intake diary panel 2011–2013 and Nutrient Intake databases. Statistical confidence level set at 95% for two–tailed *t* test. *p*-Value <0.025% considered significant. RAE, Retinol Activity Equivalents.

### 3.4. Canned Food Days vs. Non-Canned Food Days

Further analysis was performed to determine the differences in nutrient intake between the ICU and FCU groups on days when canned foods were or were not consumed. The results showed that on days when canned foods were consumed, a significantly higher percentage of people in FCU group met or exceeded the RDA for 15 different nutrients, compared to the ICU group (Table 3). Additionally, when compared to the ICU group, a significantly higher percentage of the FCU group also met or exceeded the RDA for the same 15 nutrients on days when canned foods were not consumed. Some of the largest differences in nutrient intake between the FCU and ICU groups were for vitamin A (17% higher on canned food days; 10% higher on non-canned food days); vitamin C (17% higher on canned food days; 15% higher on non-canned food days); folate (15.4% higher on canned food days; 9.9% higher on non-canned food days); vitamin B6 (13.8% higher on canned food days; 8.8% higher on non-canned food days); and calcium (12.8% higher on canned food days; 8.5% higher on non-canned food days). Although the percentage of people meeting or exceeding the RDA for dietary fiber intake did not significantly differ between the ICU and FCU groups on either canned or non-canned days, there were nearly three times as many people in the FCU group who met or exceeded the requirement on days in which canned foods were consumed, compared to the ICU group (9.4% *vs.* 3.9%, respectively).

**Table 3:** Percentage of Canned *vs.* Non-Canned consumption days in which participants met or exceeded the Recommended Dietary Allowance (RDA) for a nutrient among Frequent and Infrequent Canned Food Users.

(ICU, *n* = 2732; FCU, *n* = 2584)						
Variable	% Canned Days that the RDA Was Met or Exceeded			% Non-Canned Days that the RDA Was Met or Exceeded		
	IFU	FCU	*p*-Value	IFU	FCU	*p*-Value
Fiber	3.9	9.4	=0.04	3.0	5.5	=0.15
Protein	86.1	91.6	<0.01	85.8	89.1	<0.01
Vitamin A	31.7	48.7	<0.01	31.9	41.9	<0.01
Vitamin E	3.5	7.5	=0.13	4.2	7.7	=0.04
Vitamin C	41.0	58.0	<0.01	41.0	56.0	<0.01
Thiamin	76.3	87.2	<0.01	79.3	86.5	<0.01
Riboflavin	87.5	94.1	<0.01	88.2	93.4	<0.01
Niacin	80.5	89.5	<0.01	82.0	87.1	<0.01
Vitamin B6	64.2	78.0	<0.01	63.7	72.5	<0.01
Folate	42.1	57.5	<0.01	46.3	56.2	<0.01
Vitamin B12	82.5	90.2	<0.01	82.7	89.1	<0.01
Calcium	30.3	43.1	<0.01	31.7	40.2	<0.01
Magnesium	17.6	29.3	<0.01	18.2	25.4	<0.01
Phosphorus	85.7	92.0	<0.01	85.5	90.1	<0.01
Iron	71.0	78.4	<0.01	68.5	74.7	<0.01
Zinc	64.2	72.4	<0.01	60.3	66.6	<0.01
Selenium	91.7	94.5	<0.01	92.2	94.7	<0.01

Percentage of infrequent (*n* = 2732) and frequent (*n* = 2584) canned food users (ICU and FCU, respectively) survey days in which the Recommended Dietary Allowance (RDA) was met or exceeded for a nutrient. Data divided by days in which canned foods were consumed and days in which no canned foods were consumed. Data sourced from the NPD Group’s National Eating Trends^®^ intake diary panel 2011–2013 and Nutrient Intake databases. Statistical confidence level set at 95% for two-tailed *t* test. *p*-Value < 0.025% considered significant.

## 4. Discussion

The data show that in an average two-week period, 8 out of 10 Americans surveyed consumed at least one canned food. Of those 80% of Americans who consumed canned foods, the average individual partook in approximately five canned food eating occasions in those two weeks, or, roughly one canned food every three days. This analysis, however, did not focus on average can users (35.1% of the initial population surveyed) but instead focused on canned food users in the upper and lower tertiles of US canned food consumption in order to determine how the most frequent and least frequent of canned food consumers compared nutritionally to each other.

The results of this study indicate that more frequent canned food consumption is associated with healthier eating patterns, such as greater intake of several DGA recommended food groups and higher daily nutrient intakes. This analysis provides evidence that frequent canned food consumers took in more nutrient-dense foods such as fruits, vegetables and legumes, when compared to infrequent canned food consumers (Figure 1). The FCU group also ingested significantly higher amounts of fiber and protein, and 17 different essential micronutrients over the two-week study period (Table 2). These results are promising for health and nutrition professionals and nutrition education programs which have often been hesitant to recognize canned foods as nutritionally robust options for meeting dietary requirements. However, the overall results regarding nutrient intakes for both the ICU and FCU study groups—and for Americans in general—are still far from the ideal amounts recommended by the DGA.

Many Americans have the means to follow the recommendations in the 2010 DGA if they choose to do so, but the current US overweight and obesity statistics along with past National Health and Nutrition Examination Survey (NHANES) data show that the majority of Americans are either not aware of the DGA recommendations, or simply do not understand or conform to them [9,10]. Additionally, the federal recommendations to consume more nutrient-dense foods and less calorie-dense/nutrient-poor foods pose a challenge to a substantial number of Americans who do not have the means to do so (*i.e.*, for reasons having to do with cost and/or accessibility). For example, over 14% Americans receive assistance from the SNAP program [11], but, even with this assistance, they still tend to have lower diet quality scores than non-SNAP participants with similar incomes [12]. Furthermore, 7% of Americans, or roughly 23.5 million people live in urban or rural areas known as “food deserts,” which have limited access to fresh, healthy and affordable food [13]. Therefore, several DGA recommended items such as fresh produce or fresh seafood are not always available options for many Americans. However, canned foods are a way to get many of these recommended foods and their unique array of nutrients at affordable prices, even in underserved areas such as food deserts.

Relative to the ICU group, the FCU group were twice as likely to be participants in the government assistance programs—SNAP (11.9% *vs*. 23.5%, respectively) and WIC (6.3% *vs.* 11.8%, respectively) —highlighting the important role canned foods play for individuals facing economic hardships and who have less access to fresh and frozen food varieties. The percentage of participants in the WIC program did not reach significant difference between ICU and FCU groups in this study (Table 1), but this lack of a difference is only due to the fact that children under 6 years old (*i.e.*, all infants and a large percentage of children) were not included in the final analysis. In fact, canned fruits, vegetables, fish and beans are all WIC-eligible foods [14], and WIC recommends low-salt and low-sugar canned and jarred foods as part of a healthy infant and toddler feeding program [15].

A 2012 study by Kapica *et al.*, provided evidence that canned foods, including vegetables, fruits, fish and beans actually provided nutrients at a lower or comparable cost compared to fresh, frozen or dried options [5], showing that cost-effective, accessible, and nutrient-dense food options can be available in some of the lowest income and highest food insecure areas in the US. While fresh produce and seafood are often perceived as some of the healthiest dietary options [3], the 2010 DGA repetitively suggests that fresh, frozen and canned vegetables and fruits all count towards the DGA recommendations [2]. Additionally, canned or tinned seafood such as albacore tuna, anchovies, sardines, and salmon are all rich sources of the omega-3 fatty acids eicosapentaenoic acid (EPA) and docosahexaenoic acid (DHA), which are recommended by DGA, especially for pregnant and breastfeeding mothers [2]. Canned seafood is also a source of three of the four DGA “nutrients of concern”: with canned sardines being a good source of calcium; canned salmon, sardines and tuna being good sources of vitamin D; and canned clams containing 542 mg of potassium per serving [2].

While potassium is designated as a “nutrient of concern” by the DGA because it is a shortfall nutrient in the US diet, sodium is considered a “nutrient of concern” of because it is generally over consumed in the US diet. The DGA aims to reduce dietary sodium intake in the US diet since excess amounts can negatively affect several chronic diseases such as hypertension, diabetes and kidney disease [2]. Canned foods are often associated with sodium intake, but according to the Centers for Disease Control and Prevention (CDC), canned foods such as fruits and vegetables are not one of the top ten sources of sodium in the US diet [16]. Additionally, nutrient-dense canned foods such as fruits, vegetables and legumes come in many low-sodium and sodium-free options. Moreover, studies have shown that simply rinsing off sodium-containing canned foods such as vegetables and beans can reduce sodium content by up to 41% [17]. The results from this study show that the FCU group consumed more sodium than the ICU group (Table 2), however the FCU group also consumed more potassium as well; and the difference in potassium intake between groups was greater than for sodium intake (~19% *vs.* ~12%, respectively). The sodium/potassium ratio was also 7% lower in the group which consumed canned foods more frequently (1.49 for ICU *vs.* 1.39 for FCU). The sodium/potassium ratio has been shown to be a more representative indicator of cardiovascular disease and hypertension risk than sodium intake levels alone [18,19], with increased potassium levels being shown to be able to diminish the negative effects of sodium on hypertension [20]. Although high sodium intakes are epidemic in the US, they are not really an issue specific to canned foods, rather they are a ubiquitous part of the US food supply and American eating patterns as a whole.

While this research has focused on canned food consumption and essential nutrient intake, canned foods are also comparable to fresh foods as a source of antioxidants and bioactive components. In 2012, Durst and Weaver provided evidence that the canning and storage process can increase the content of certain nutrients and antioxidant function in peaches after 3 months [21]. This phenomenon likely occurs during processing in which the inactivation of degradation enzymes inhibits the breakdown of ascorbic acid and other antioxidants. The researchers showed that vitamin C, folate and antioxidant levels as measured by ferric reducing antioxidant power (FRAP) assay were all higher when compared to fresh peaches. Importantly, the measurements showed that folate, vitamin C, and antioxidants levels were stable over the three months storage period in the canned peaches, and most alterations in nutrient content occurred during processing and not over the storage period. So, while the thermal treatment used in the canning process can result in the breakdown or transformation of some water-soluble vitamins, the remaining nutrients are much more stable over time compared to those in fresh or frozen products since there is less exposure to oxidation and microbial threats [6,7]. Recent research has even shown that the thermal processing techniques used in the canning process may actually preserve or enhance some nutritional qualities in nutrient-dense foods such as canned beans, vegetables and fruits when compared to fresh foods [4,22,23,24].

Generally, fresh and canned foods contain similar quantities of most fat-soluble vitamins, minerals and fiber. Although, there is evidence that some nutrient and bioactive losses occur during the canning process, there is also evidence to the contrary, therefore it is important to recognize that the consumption of nutrient-dense canned foods are a viable and cost effective way to contribute to the overall nutritional quality of the diet [6,7]. The canning process may even increase the bioavailable levels of certain carotenoids with the best known example being the increase in lycopene in processed tomato products [4]. Additionally, processed tomatoes have higher β-carotene levels compared to fresh tomatoes, as do a variety of other fruits and vegetables which can range up to 50% higher than their fresh counterparts [25]. Furthermore, the canning process has also been shown to improve the protein (>7%) and fiber (>5%) content of multiple bean varieties, while also reducing the quantity of lectins [22]. While the canning process can improve the nutrient and bioactive levels of certain foods, it is also been shown to reduce the levels of certain nutrients in some foods as well [21,22]. Therefore, the canning process likely affects all foods differently, and the changes in overall nutrient and bioactive profile cannot be fully extrapolated from the currently available body of science. Further research is needed on different canning processes, storage times, and the resultant nutrient and bioactive profiles of canned foods in order to better understand how these foods affect the overall nutritional intake of their consumers.

There are several strengths to this analysis. For one, it is based on a large, nationally representative sample including both children and adults. Additionally, the data are drawn from two-week averages instead of single-day recall data. This study is limited in scope since it was an observational study; therefore the causality of the observed effects cannot be conclusively determined. As with all dietary survey data, the accuracy of the estimates are dependent on the accuracy of survey respondents. Finally, while canned food use is fairly easy for individuals to determine at home cooked meals, they are not as easy to identify when eating out or away from home. Therefore, misidentification and potential underestimation of canned food use away from home may have skewed the data towards lower canned food use in populations who frequently consume meals away from home.

## 5. Conclusions

In summary, this analysis indicates that children and adults in the US who frequently consume canned foods, have higher nutrient intakes and healthier eating habits compared to those who infrequently consume canned foods. In this study, frequent canned food consumption was associated with higher intakes of 17 essential nutrients including calcium, potassium and fiber—three shortfall nutrients according to the 2010 Dietary Guidelines for Americans. Frequent canned food consumption was also associated with a greater likelihood of consuming nutrient-dense food groups recommended by the Dietary Guidelines for Americans such as: fruits, vegetables, dairy products and protein foods. Therefore, in addition to fresh foods, nutrient-dense canned foods should be promoted by health and nutrition professionals for their ability to improve nutrient intakes and the diet quality of Americans.
